# PNG-Net: Physics Noise-Guided Network for Infrared Image Nonuniformity Correction

**DOI:** 10.3390/s26196021

**Published:** 2026-09-23

**Authors:** Mei Ming, Haifeng Zhang, Hongyuan Wang

**Affiliations:** 1 School of Astronautics, Harbin Institute of Technology, Harbin 150001, China; mingmei_hit@163.com (M.M.); zhanghf@hit.edu.cn (H.Z.); 2 Zhengzhou Research Institute, Harbin Institute of Technology, Zhengzhou 450000, China

**Keywords:** infrared image denoising, nonuniformity correction, PSD-like spectral noise prior, frequency-domain analysis, noise-guided residual learning

## Abstract

**Highlights:**

**What are the main findings?**

- A physics-guided NUC network (PNG-Net) is proposed for infrared FPA images.
- Frequency-band masks capture FPN and striping; noise gating preserves edges.

**What are the implications of the main findings?**

- PNG-Net improves non-uniformity correction for infrared FPA imaging.
- It preserves edges and thermal gradients for downstream infrared tasks.

**Abstract:**

Infrared thermal imaging is widely used for industrial fault diagnosis because it enables non-contact measurements and reveals temperature anomalies. However, detector nonuniformity degrades image quality and can affect subsequent analysis. Existing nonuniformity correction (NUC) methods face a trade-off between suppressing structured noise and preserving fine image details. To address this issue, a Physics Noise-Guided Network (PNG-Net) is proposed for infrared image nonuniformity correction. First, the input image is decomposed into low-, mid-, and high-frequency components using the fast Fourier transform (FFT), and multi-frequency information is adaptively integrated through a dynamic frequency-band selection module. Second, a learnable PSD-like noise estimator derives a spectral noise prior from the Fourier magnitude spectrum, and an anisotropic mask adds directional information about stripe noise. During feature restoration, the estimated prior noise is incorporated into the proposed Noise-Guided Stack to conditionally modulate multi-scale residual features, thereby progressively recovering structural details and thermal gradients through hierarchical encoder–decoder skip connections. Finally, global residual learning is adopted to predict the nonuniformity correction residual. On the synthetic infrared nonuniformity dataset, PNG-Net achieves a PSNR of 48.92 dB and an SSIM of 0.990, outperforming the compared methods in these two metrics.

## 1. Introduction

Motors are key components in industrial equipment, rail transit, electric vehicles, and aerospace systems. Their condition affects operational continuity, equipment safety, and maintenance costs. Faults such as bearing wear, rotor misalignment, broken rotor bars, winding short circuits, and insulation aging can alter heat generation and the motor’s surface temperature distribution. Infrared (IR) images of the motor housing, bearing end caps, and regions adjacent to the windings can therefore provide useful information for non-contact condition monitoring [1,2,3].

In real industrial environments, however, the acquired IR images are severely contaminated by nonuniformity (NU) noise due to factors such as the response nonuniformity of infrared focal plane array (IRFPA) detectors based on materials such as mercury cadmium telluride, vanadium oxide, and amorphous silicon, inconsistencies in readout circuitry, and ambient temperature drift [4,5,6,7]. This noise typically manifests as fixed-pattern noise (FPN), striping noise, and slowly time-varying 1/f flicker noise. It not only degrades the contrast and temperature measurement accuracy of thermal images but also obscures critical thermal anomaly signatures such as localized hot spots and temperature gradient variations, thereby compromising the subsequent accuracy of fault feature extraction, state recognition, and fault diagnosis.

Conventional NUC methods are broadly classified as calibration-based or scene-based [7,8]. Calibration-based methods may require recalibration as detector responses drift with temperature, operating time, or aging. Scene-based methods depend on assumptions about scene variation and, in some cases, motion estimation. Violations of these assumptions can cause ghosting, residual patterns, or slow convergence [8,9].

In recent years, deep learning has substantially advanced image restoration techniques. Transformer-based models such as IPT [10], SwinIR [11], Uformer [12], and Restormer [13] have enhanced the modeling capability for long-range dependencies and multi-scale degradations through pre-training, window-based attention, hierarchical encoder–decoder architectures, and multi-DConv head transposed attention mechanisms. MAXIM [14], AirNet [15], and NAFNet [16] have further improved general image restoration performance from the perspectives of multi-axis feature mixing, unknown degradation modeling, and lightweight non-linear modeling. HAT expands the effective receptive field via channel attention, window-based self-attention, and overlapping cross-attention, while FFTformer leverages Fourier-domain multiplication to construct efficient frequency-domain attention, providing novel global modeling paradigms for high-resolution image restoration [17,18]. These works validate the effectiveness of Transformers and frequency-domain operations in image denoising, deblurring, and enhancement tasks.

However, most existing methods directly borrow visible-light image denoising models and treat IR thermal image restoration as a generic image reconstruction problem, while overlooking the formation mechanisms and frequency-domain distribution characteristics of IR NU noise. Consequently, these approaches tend to produce residual striping, over-smoothing of hot spots, and loss of subtle thermal anomaly details in complex industrial scenarios, rendering them inadequate for high-precision motor fault detection. In fact, IR NU noise exhibits exploitable statistical regularities in the frequency domain. Fixed-pattern noise, gain drift, and slowly varying background offsets typically concentrate energy in the low-frequency region. Vertical stripes arising from column-wise detector readout differences manifest as concentrated spectral lines orthogonal to the stripe orientation in the two-dimensional spectrum. The power spectrum of 1/f noise decays with increasing frequency. In contrast, the motor housing contours, heat-sink edges, bearing end caps, and localized abnormal hot spots simultaneously contain low-frequency thermal backgrounds, mid-frequency regional structures, and high-frequency edge gradients. Therefore, simply attenuating low-frequency or directional frequency components inevitably destroys the true temperature structures; NU noise must be adaptively suppressed based on noise statistics rather than being confined to fixed frequency bands [9].

Motivated by the above analysis, we propose PNG-Net, a physics-guided network for infrared image NUC. The network combines frequency decomposition, a learned spectral noise prior, and residual restoration. Its main contributions are as follows.

(1) A physics-guided nonuniformity correction network, PNG-Net, is proposed, which explicitly incorporates the IRFPA gain–offset response model and the spectral priors of fixed-pattern noise and 1/f noise into the network design. This establishes a unified formulation that combines physical noise modeling with deep learning-based restoration, rather than treating IR NUC as a generic image restoration problem.

(2) A frequency-aware noise modeling strategy combines FFT-based frequency decomposition, dynamic frequency-band selection, a PSD-like noise estimator, and anisotropic masking. The resulting spectral noise prior describes noise-related frequency patterns and directional stripe components.

(3) A noise-guided residual stack uses the learned spectral noise prior to condition multi-scale feature restoration. Through a noise-gating mechanism, the feature responses at different spatial locations are adaptively modulated. This approach effectively suppresses structured noise while preserving image edges, temperature gradients, and weak texture details.

## 2. Related Work

### 2.1. Deep Learning-Based Image Restoration Methods

Early deep learning-based image restoration primarily relied on local feature extraction via convolutional networks and multi-scale encoder–decoder architectures. MPRNet employed a multi-stage progressive restoration scheme with supervised attention, propagating contextual and high-resolution information across stages [19]. MIRNet-v2 leveraged parallel multi-resolution branches and feature exchange mechanisms to balance local details and global context [20]. MIMO-UNet reduced the computational overhead of coarse-to-fine restoration through a multi-input multi-output structure [21]. DGUNet unfolded the proximal gradient descent procedure into a trainable network, handling complex degradations while retaining a certain degree of model interpretability [22]. These works laid the foundation for multi-scale detail recovery; however, the limited receptive fields of convolutional operators restrict their capability to model long-range striping patterns and global low-frequency biases.

Transformers have introduced a new technical pathway for high-resolution image restoration by establishing long-range feature correlations via self-attention. IPT utilized large-scale pre-training on diverse degradation data to handle denoising, super-resolution, and deraining tasks in a unified manner [10]. SwinIR adopted shifted-window Transformers to achieve local attention and cross-window information propagation [11]. Uformer embedded locally enhanced window attention into a U-shaped encoder–decoder architecture [12]. Restormer transposed attention computation from the spatial dimension to the channel dimension and combined it with a gated depthwise convolutional feed-forward network, enabling efficient processing of high-resolution images with lower complexity [13]. MAXIM modeled local and global spatial interactions via multi-axis gated MLPs [14], while NAFNet demonstrated that complex non-linear activations are not essential for achieving high restoration performance [16]. Although these methods perform well on generic restoration tasks, their degradation models are typically learned implicitly from data and lack explicit physical constraints tailored to IRFPA nonuniformity.

For unknown or mixed degradations, AirNet employed contrastive learning to extract degradation representations that drive a unified restoration network [15]. PromptIR utilized learnable prompts to encode different degradation types and intensities [23]. U2-Former enhanced the decoupling of noise from background through nested U-shaped structures and multilayer contrastive learning [24]. These approaches improve model adaptability to composite degradations; however, for IR NU noise, learning only abstract degradation embeddings remains insufficient to interpret the suppression behavior across different frequencies and orientations, nor can it guarantee that localized motor hot spots are not erroneously removed.

HAT combined channel attention, window attention, and overlapping cross-attention to expand the effective input region utilized by the restoration network [17]. Stripformer constructed strip tokens along horizontal and vertical directions, exhibiting strong representational capability for directional degradations [25]. Retinexformer guided Transformer-based restoration of low-light degradations through a physical illumination model [26], demonstrating that incorporating imaging physics as an attention condition enhances the specificity of the restoration process. The present work draws inspiration from these efficient attention mechanisms and physics-guided principles, yet targets IR NU images characterized by gain–offset errors, 1/f spectra, and row–column directional dependencies, rather than generic low-light or motion blur.

### 2.2. Frequency-Domain Analysis-Based Image Restoration Methods

Frequency-domain analysis is an essential tool for understanding and processing IR NU noise. Studies have shown that FPN predominantly resides in low-frequency components, with its power spectral density concentrated in the low-frequency region, whereas the PSD of 1/f noise decays inversely with frequency and exhibits specific directional distributions. These frequency-domain characteristics provide a theoretical foundation for designing targeted denoising algorithms. In recent years, several works have begun to incorporate frequency-domain information into deep learning-based denoising networks. For instance, FFC integrated fast Fourier transforms into convolution operations to achieve a global receptive field [27]. LaMa employed fast Fourier convolutions to obtain image-level receptive fields, validating the effectiveness of frequency-domain operations in high-resolution structure modeling [28]. FFTformer, based on the convolution theorem, transformed spatial-domain correlation operations into element-wise frequency-domain multiplications and selected which high- and low-frequency information to retain via a frequency discriminative feed-forward network [18]. These works indicate that frequency-domain representations can reduce the cost of global interactions and enhance model responsiveness to long-range degradations.

However, most existing frequency-domain restoration methods treat the Fourier transform merely as an efficient tool for global feature mixing, without explicitly estimating the noise PSD specific to a given imaging system. For IR images, the low-frequency region contains both gain and offset nonuniformities and the overall temperature distribution of the motor; the high-frequency region includes both random noise and hot-spot edges as well as heat-sink textures. Therefore, simple filtering based on fixed frequency thresholds cannot reliably distinguish noise from fault-related information.

## 3. Methodology

### 3.1. Problem Formulation and Motivation

The nonuniformity noise in infrared images primarily arises from inconsistent responses across individual detector elements in the focal plane array. Let the ideal noise-free image be denoted as $I_{\mathrm{clean}}$ and the observed noisy image as $I_{\mathrm{noisy}}$. The imaging model can be expressed as:

(1) $$I_{\mathrm{noisy}}(x,y,t)=\left[1+\Delta G(x,y)\right]\cdot I_{\mathrm{clean}}(x,y,t)+O(x,y)+N_{s}(x,y,t)+N_{1/f}(x,y,t)+N_{g}(x,y,t)$$

where ΔG(x,y) denotes gain nonuniformity, O(x,y) offset nonuniformity noise, $N_{s}(x,y,t)$ stripe noise, $N_{1/f}(x,y,t)$ 1/f noise, and $N_{g}(x,y,t)$ Gaussian noise.

Fixed-pattern noise (FPN) is modeled as a combination of multiplicative gain fluctuation and additive offset fluctuation, defined as:

(2) $$N_{\mathrm{FPN}}(x,y)=\Delta G(x,y)I_{\mathrm{clean}}(x,y,t)+O(x,y)$$

where the gain fluctuation ΔG and offset fluctuation O are sampled from a zero-mean Gaussian distribution:

(3) $$\left\{\begin{array}{l} \Delta G∼N\left(\begin{array}{cc} 0, & \sigma_{g}^{2} \end{array}\right)\\ O∼N\left(\begin{array}{cc} 0, & \sigma_{o}^{2} \end{array}\right) \end{array}\right.$$

Traditional methods generally assume that the gain term ΔG and the offset term *O* vary slowly or remain constant across the spatial domain; however, practical scenarios present considerably more complex distributions.

The stripe noise usually originates from inconsistent response of row/column readout channels. The most common type of noise in infrared focal plane arrays is column stripe noise. For column stripe $N_{s}(x,y)=N_{s}(x)$, each column has a random bias:

(4) $$N_{s}(x)∼N(\begin{array}{cc} 0, & \sigma_{s}^{2} \end{array})$$

The 1/f noise is generated in the frequency domain, and its PSD is defined as:

(5) $$S_{1/f}(f_{x},f_{y})=\frac{K}{{\left(\sqrt{{f_{x}}^{2}+{f_{y}}^{2}}+f_{0}\right)}^{\alpha }}$$

where K represents the noise intensity; $f_{0}$ represents low-frequency cutoff or regularization term; α represents power-law exponent; Ideal 1/f noise typically has α≈1. The 1/f noise can generally be obtained by first generating white noise and then filtering it in the frequency domain. White Gaussian noise w is defined as:

(6) $$w(x,y)∼N(\begin{array}{cc} 0, & 1 \end{array})$$

Then, its Fourier spectrum was filtered by a frequency-dependent amplitude response:

(7) $$W(f_{x},f_{y})=F\{w(x,y)\}$$

The PSD of white noise is approximately a constant. Therefore, the filter *H* is defined as $H(f_{x},f_{y})=\sqrt{S_{1/f}(f_{x},f_{y})}$, and 1/f noise is defined as:

(8) $$n_{1/f}(x,y)=F^{-1}\left(N_{1/f}(f_{x},f_{y})\right),\;\mathrm{where}\;N_{1/f}(f_{x},f_{y})=H(f_{x},f_{y})W(f_{x},f_{y})$$

After inverse Fourier transform, the 1/f noise was normalized to zero mean and unit standard deviation, then rescaled to the target noise level:

(9) $$N_{1/f}(x,y)=\sigma_{1/f}\frac{n_{1/f}(x,y)-\mu }{\sigma }$$

The Gaussian noise $N_{g}(x,y)$ is defined as a zero-mean Gaussian distribution:

(10) $$N_{g}(x,y)∼N(\begin{array}{cc} 0, & \sigma_{g}^{2} \end{array})$$

Conventional deep learning-based denoising models primarily operate in the spatial domain, employing convolutional operations or self-attention mechanisms to learn the degradation and restoration mappings. Nevertheless, NU noise generated by IR detectors often exhibits global correlations, directional consistency, and cross-scale distribution characteristics. In particular, column-wise striping noise and FPN frequently manifest as structural features visually similar to genuine object edges in the spatial domain, making it difficult for networks to reliably distinguish between noise and valid textural information. Consequently, relying solely on spatial-domain feature learning inevitably encounters a trade-off: enhancing the denoising capability tends to blur object boundaries, whereas preserving fine details leaves substantial residual noise.

In contrast to the spatial domain, IR NU noise exhibits more stable and physically meaningful distribution regularities in the frequency domain. Specifically: (1) FPN, as a slowly varying or approximately periodic noise component in space, concentrates its spectral energy primarily in the low-frequency region; (2) the power spectral density (PSD) of 1/f noise decays with increasing frequency and tends to exhibit pronounced energy aggregation along specific orientations, such as the row or column directions; (3) genuine scene targets contain rich structural and textural information, with their spectral distributions typically spanning low-, mid-, and high-frequency bands in a relatively dispersed manner. As illustrated in Figure 1, compared with clean images, noisy images exhibit significantly stronger energy concentration in the central region of the spectrum, particularly within low-frequency bands and specific directional frequency components. This observation indicates that the frequency domain effectively characterizes the physical signatures of IR NU noise, providing a critical basis for noise identification and suppression.

These observations motivate two aspects of the proposed design:

(1) Frequency Aggregation. FPN and striping noise tend to concentrate in the low-frequency region or specific directional frequency bands within the 2D Fourier spectrum, whereas genuine target information exhibits a substantially broader spectral distribution.

(2) Frequency Decoupling. Owing to the significant disparity between the energy distributions of noise and true targets, the noise component can be explicitly estimated in the frequency domain and subsequently incorporated as prior information into feature extraction and attention computation. This strategy suppresses the propagation of noise-related features through the network and enhances the disentanglement of noise from target information.

PNG-Net combines these ideas in an encoder–decoder architecture. Frequency decomposition provides low-, mid-, and high-frequency components for adaptive feature fusion. A PSD-like noise estimator and an anisotropic mask produce a spectral noise prior, which conditions the noise-guided residual stack at multiple scales. The overall architecture of PNG-Net is illustrated in Figure 2.

### 3.2. Network Architecture Design

#### 3.2.1. Frequency Decomposition and Dynamic Selection

To effectively disentangle noise from signal components, PNG-Net first transforms the input image from the spatial domain to the frequency domain and decomposes it into different frequency bands. Given an input image $I\in {\mathbb{R}}^{C\times H\times W}$, its frequency-domain representation is obtained via the fast Fourier transform (FFT):

(11) $$X=F(I)=\sum_{x=0}^{H-1}\sum_{y=0}^{W-1}I(x,y)e^{-j2\pi \left(\frac{ux}{H}+\frac{vy}{W}\right)}$$

To separate the background structures from stochastic high-frequency interference, we construct frequency decomposition masks based on the centered spectral radius $r(u,v)=\sqrt{{u^{\prime }}^{2}+{v^{\prime }}^{2}}$, where $\left(u^{\prime },v^{\prime }\right)$ denote the shifted frequency-domain coordinates. Let $R_{L}$ and $R_{H}$ be the cutoff ratios for the low- and high-frequency radii, respectively. The low-frequency mask $M_{low}$, mid-frequency mask $M_{mid}$, and high-frequency mask $M_{high}$ are defined as:

(12) $$M_{low}(u,v)=I(r(u,v)\leq R_{L}\cdot r_{max})$$

(13) $$M_{mid}(u,v)=I(R_{L}\cdot r_{max}<r(u,v)\leq R_{H}\cdot r_{max})$$

(14) $$M_{high}(u,v)=I(r(u,v)>R_{H}\cdot r_{max})$$

where I(⋅) is the indicator function and $r_{max}$ denotes the maximum spectral radius. The default cutoff ratios are $R_{L}$ = 0.125 and $R_{H}$ = 0.5. Applying these masks to the frequency representation and performing inverse 2D FFT yields the spatially decoupled low-, mid-, and high-frequency images:

(15) tlow=Re(F−1(X⋅Mlow))

(16) tmid=Re(F−1(X⋅Mmid))

(17) thigh=Re(F−1(X⋅Mhigh))

where $F^{-1}(\cdot )$ denotes the inverse FFT. The low-frequency image primarily contains the background and slowly varying NU noise; the mid-frequency image preserves the main structural information of the target; and the high-frequency image captures textural details and stochastic noise. This decomposition enables differentiated processing strategies for distinct frequency components in subsequent stages.

To adaptively fuse the extracted frequency components, we construct a dynamic frequency-band selector. The multi-channel frequency components $f_{low},f_{mid},f_{high}\in {\mathbb{R}}^{C\times H\times W}$, obtained after feature projection from ($t_{low}$, $t_{mid}$, $t_{high}$), are concatenated and fed into a lightweight channel-aware MLP to generate dynamic aggregation weights:

(18) $$g=[\mathrm{GAP}(f_{low})∥\mathrm{GAP}(f_{mid})∥\mathrm{GAP}(f_{high})]\in {\mathbb{R}}^{3C\times 1\times 1}$$

(19) $$w=\mathrm{Softmax}(W_{2}*\mathrm{GELU}(W_{1}*g))\in {\mathbb{R}}^{3\times 1\times 1}$$

where GAP denotes global average pooling and ∥ indicates channel-wise concatenation. The resulting fused frequency-domain feature $f_{fused}$ is computed as:

(20) $$f_{fused}=w_{0}\cdot f_{low}+w_{1}\cdot f_{mid}+w_{2}\cdot f_{high}$$

The final feature fed into the encoder–decoder backbone is expressed as:

(21) $$F_{0}=feat+w_{fuse}\cdot f_{fused}+w_{high}\cdot f_{high}$$

where feat denotes the shallow feature extraction layer, $w_{fuse}$ and $w_{high}$ represent the fused frequency-domain feature and the high-frequency feature weight, with initial weight values of 0.6 and 0.2, respectively. The feature weights are automatically optimized during the network training process.

#### 3.2.2. PSD-like Noise Estimator and Anisotropic Masking

The PSD-like noise estimator learns a spectral noise prior from the input Fourier magnitude spectrum. This prior is a learned descriptor, not a calibrated measurement of physical noise power. In contrast, the reference noise PSD used for evaluation is estimated from the squared Fourier magnitude of a known noise residual. The anisotropic mask supplements the learned prior with directional information about stripe noise.

A three-layer convolutional network directly predicts the isotropic PSD-like noise from the 2D FFT amplitude spectrum of the input image:

(22) $$P_{raw}=\sigma (W_{3}*\mathrm{ReLU}(W_{2}*\mathrm{ReLU}(W_{1}*|X|)))$$

where |X| is the Fourier amplitude spectrum, and σ(⋅) is the Sigmoid activation function.

To address the strongly directional horizontal and vertical striping patterns prevalent in IR images, we construct a polar-coordinate directional mask via a set of learnable affine projection parameters:

(23) $$m_{horz}(u,v)=\sigma (W_{horz}\cdot {\left[u_{norm},v_{norm}\right]}^{T}+b_{horz})$$

(24) $$m_{vert}(u,v)=\sigma (W_{vert}\cdot {\left[u_{norm},v_{norm}\right]}^{T}+b_{vert})$$

where $\left[u_{norm},v_{norm}\right]$ are the normalized centered frequency coordinates. Learnable weight parameters α,β∈ℝ are introduced to form the final directional mask $M_{dir}$.

(25) $$M_{dir}=\sigma (\alpha )\cdot m_{horz}+\sigma (\beta )\cdot m_{vert}$$

The directional physical PSD-like noise is formulated as:

(26) $$P_{dir}=P_{raw}⊙M_{dir}$$

where ⊙ denotes the Hadamard product.

To enable adaptive physical-prior regularization at different stages of the U-Net architecture, the estimated $P_{dir}$ down-sampled via multi-scale average pooling, yielding noise maps $P_{1}$, $P_{2}$, and $P_{3}$ that match the resolutions of the respective encoder levels. The resampling of spectral noise prior $P_{dir}$ does not assume a direct coordinate correspondence between a frequency coordinate (u, v) and a spatial location (x, y). Instead, the prior transfers global spectral energy and orientation statistics to the spatial residual blocks as a conditioning signal. The anisotropic mask further encodes row- and column-oriented stripe tendencies, and the subsequent convolutional residual blocks learn how this frequency-conditioned descriptor should modulate spatial features at each scale.

#### 3.2.3. Noise-Guided Residual Stack

To fully exploit the complementary information from both frequency and spatial domains while maintaining efficiency and stability for high-resolution restoration, PNG-Net introduces the Noise-Guided Stack, which consists of multiple sequentially stacked noise-guided residual blocks.

(1) Noise-Guided Residual Block

Let the input feature be $X\in {\mathbb{R}}^{B\times C\times H\times W}$, and the corresponding noise prior map obtained from the PSD-like noise estimator be $P\in {\mathbb{R}}^{B\times 1\times H\times W}$. First, a baseline residual mapping is constructed using two convolutional layers:

(27) $$R=F_{r}(X)=W2*\delta \left(W1*X\right)$$

where * denotes the convolution operation. δ(⋅) is the ReLU activation function.

Subsequently, the single-channel noise prior is projected to the same channel dimension as the input features via a 1×1 convolution, and a noise-gating map G is generated using the Sigmoid function:

(28) $$G=\sigma \left(W_{g}*P\right)$$

where $G\in {\mathbb{R}}^{B\times C\times H\times W}$ reflects the relative degree of noise contamination at different spatial locations and feature channels.

The residual feature is then modulated by the gating map:

(29) $$\tilde{R}=R⊙\left(1+G\right)$$

Finally, the output of the noise-guided residual block is formulated as $X+\tilde{R}$. Since G is produced by a Sigmoid activation, G lies in (0, 1), and the multiplicative residual factor 1+G lies in (1, 2). Consequently, the gate does not attenuate the residual branch; instead, it adaptively amplifies the learned correction residual in channels and regions indicated by the spectral noise prior. This design ensures that in noise-dominant regions, stronger gating leads to more effective noise suppression, while in low-noise regions, the limited amplification preserves structural details.

(2) Multilayer Noise-Guided Residual Stack

Several noise-guided residual blocks are stacked to model mixed degradations at successive feature-processing stages. If $X^{\left(l\right)}$ is the input to the *l*-th block, its output is:

(30) $$X^{\left(l\right)}+F_{r}^{\left(l\right)}\left(X^{\left(l\right)}\right)⊙\left[1+G^{\left(l\right)}\left(P\right)\right]$$

where $F_{r}^{\left(l\right)}(\cdot )$ denotes the residual feature extraction mapping of the *l*-th block, and $G^{\left(l\right)}(\cdot )$ denotes the corresponding noise prior gating mapping.

Although all residual blocks share the same noise prior, their convolutional and gating parameters are independently learnable, allowing different layers to capture distinct noise response patterns. The shallower blocks primarily suppress prominent FPN and striping responses; the intermediate blocks further correct local background fluctuations and weak periodic noise; and the deeper blocks focus on restoring edges and textures that have been corrupted by noise. In this manner, the Noise-Guided Stack forms a progressive, coarse-to-fine noise correction process.

#### 3.2.4. Skip-Connection Fusion Module

In the U-Net backbone, directly adding or simply concatenating the encoder’s shallow features with the decoder’s upsampled deep features in the skip connections can readily cause geometric structural misalignment, due to spatial sub-pixel shifts introduced by upsampling and the directional polarization of IR striping. To address this issue, we design a Fusion Block that incorporates Channel-Coordinate Attention (CCA) to reweight both branches before fusion. 

Given the skip-branch input feature $F_{skip}$ and the main-branch upsampled feature $F_{up}$. CCA is applied to each branch independently. For a feature $F\in {\mathbb{R}}^{C\times H\times W}$, one-dimensional average pooling along the horizontal and vertical axes gives:

(31) $$\left\{\begin{array}{l} F_{h}(c,h,1)=\frac{1}{W}\sum_{w=1}^{W}F(c,h,w)\\ F_{w}(c,1,w)=\frac{1}{H}\sum_{h=1}^{H}F(c,h,w) \end{array}\right.$$

The pooled axis descriptors are combined through a shared projection and used to obtain the directional attention factors $a_{h}$ and $a_{w}$:

(32) $$\left\{\begin{array}{l} a_{h}=\sigma (W_{h}*y_{h})\\ a_{w}=\sigma (W_{w}*y_{w}) \end{array}\right.$$

where $y_{h}$ and $y_{w}$ are obtained by splitting y along the spatial axis, with $y_{h}\in {\mathbb{R}}^{\frac{C}{r}\times H\times 1}$, $y_{w}\in {\mathbb{R}}^{\frac{C}{r}\times 1\times w}$, and y is defined as:

(33) $$y=\mathrm{ReLU}(W_{1}*[F_{h}∥F_{w}^{T}])\in {\mathbb{R}}^{\frac{C}{r}\times (H+W)\times 1}$$

The directional attention factors then modulate the input feature:

(34) $$\mathrm{CCA}(F)=F⊙a_{h}⊙a_{w}$$

After CCA reweighting, the two branches complete cross layer fusion through channel level concatenation and subsequent convolution feature interaction:

(35) $$F_{fuse}=W_{3\times 3}*\mathrm{GELU}(W_{1\times 1}*[\mathrm{CCA}(F_{up})∥\mathrm{CCA}(F_{skip})]))$$

### 3.3. Loss Function Design

The loss function consists of three parts: spatial domain reconstruction loss, spectral consistency loss, and perceptual loss. Among them, the spatial domain reconstruction loss is used to constrain the overall deviation between the reconstruction result and the target image, which can directly optimize pixel-level errors and improve PSNR. Spectral consistency loss constrains the spectral similarity between the output image and the clean image, thereby enhancing the ability to suppress texture and stripe noise. VGG perceptual loss aims to further constrain the corrected image to maintain consistency with the noise-free reference image in terms of high-level structure and local texture features. When only pixel-level loss is used, the model can improve PSNR, but it tends to generate overly smooth results, resulting in the weakening of thermal edges, small target contours, and subtle textures in infrared images. Although the VGG network is pre-trained on RGB natural images, its shallow and mid-level features can still extract general edge, gradient, local structure, and texture responses. Therefore, perceptual loss can compensate for the deficiency in L1 loss in maintaining visual structure, enabling the network to better preserve thermal target boundaries and detailed information while suppressing nonuniform noise.

The spatial domain reconstruction loss adopts L1 norm to reduce the joint constraint of the impact of image mutation points:

(36) $$L_{1}=\frac{1}{HW}\sum_{x=1}^{H}\sum_{y=1}^{W}\left|\hat{I}(x,y)-I_{\mathrm{gt}}(x,y)\right|$$

Spectral consistency loss constrains the spectral similarity between the output image and the clean image:

(37) $$L_{\mathrm{freq}}=\frac{1}{HW}\sum_{u=1}^{H}\sum_{v=1}^{W}\left|F(\hat{I})(u,v)-F(I_{\mathrm{gt}})(u,v)\right|$$

Perceived loss is based on the high-level features of pre-trained VGG-19 networks, which enhances visual perception realism and avoids the smooth plastic texture caused by traditional denoising networks. The method of feeding a single-channel infrared image into the RGB pre-trained VGG-19 network is to first copy the single-channel infrared image into three channels and then use ImageNet’s RGB mean and variance normalization. The shallow and mid-level features of VGG-19 mainly respond to edges, textures, and local structures, which still exist in infrared images. Although VGG-19 is pre-trained on RGB natural images, three channel replication can preserve the infrared intensity structure while meeting the input format of VGG-19.

(38) $$L_{\mathrm{percept}}=\sum_{k}\frac{1}{C_{k}H_{k}W_{k}}{\left\|\Psi_{k}(\hat{I})-\Psi_{k}(I_{\mathrm{gt}})\right\|}_{2}^{2}$$

The total loss is a weighted sum of the three terms:

(39) $$L_{\mathrm{total}}=L_{1}+\lambda_{\mathrm{freq}}L_{\mathrm{freq}}+\lambda_{\mathrm{percept}}L_{\mathrm{percept}}$$

where $\lambda_{\mathrm{freq}}$ and $\lambda_{\mathrm{percept}}$ are hyperparameters used to balance the contributions of each loss component. In this paper, $\lambda_{\mathrm{freq}}$ and $\lambda_{\mathrm{percept}}$ are both set to 0.001.

## 4. Experimental Results and Analysis

### 4.1. Experimental Setup

To validate the effectiveness of PNG-Net, comprehensive experiments were conducted on a synthetic dataset. The synthetic dataset was constructed based on a publicly available infrared motor fault dataset, which was originally created by the Electrical and Computer Engineering Laboratory at Babol Noshirvani University of Technology for industrial motor fault analysis [29]. Training and test samples were generated by injecting simulated FPN, striping noise, and 1/f noise into this dataset. The original clean images were divided before noise synthesis into 299 training images and 70 testing images, each with a resolution of 240 × 320. The corresponding noisy–clean pairs were stored separately for training and test, and no clean source image was shared between the two subsets. The train and test set contains 11 directory-defined operating/fault categories: A&B50, A&C&B10, A&C&B30, A&C10, A&C30, A10, A30, A50, Fan, Noload, and Rotor-0; the dataset introduction is shown in Table 1. The noise parameters were configured according to the statistical characteristics of real detectors; the 8-bit gray-level ranges of standard deviation settings for different noises is shown in Table 2.

For quantitative evaluation of the correction performance, Peak Signal-to-Noise Ratio (PSNR) and Structural Similarity Index Measure (SSIM) were adopted as the primary evaluation metrics. PSNR measures the pixel-wise reconstruction accuracy, while SSIM assesses the structural fidelity. PNG-Net was implemented in PyTorch 3.9 and trained on an NVIDIA GeForce RTX 3090 GPU (ASUSTeK Computer Inc., Taipei, Taiwan) using the Adam optimizer, with an initial learning rate of 1 × 10^−4^, a batch size of 8, and a total of 200 training epochs.

### 4.2. Quantitative Evaluation

We compared PNG-Net against a variety of state-of-the-art deep learning methods, including scene-based/calibration-free infrared NUC (SBNUC [5],NN-NUC [30]), CNN-based approaches (DnCNN [31], U-Net [32], MPRNet [19], NAFNet [16], AirNet [15]), Transformer-based approaches (Uformer [12], Restormer [13]), and frequency-domain methods (FFC [27], FFTformer [18]). Table 1 reports the quantitative results on the synthetic test set.

For fair comparison, these methods were retrained on the training set used in this article and evaluated on the same 70 test pairs on our NVIDIA RTX 3090 GPU, including performance evaluation, inference time evaluation, and generalization ability. These baselines were also trained for 200 iteration cycles.

Evaluation metrics for nonuniformity correction effectiveness include PSNR (Peak Signal-to-Noise Ratio), SSIM (Structural Similarity Index Measure), SNI (Stripe Noise Index), RMNU (Row Mean Nonuniformity), CMNU (Column Mean Nonuniformity) and EPI (Edge Preservation Index). PNG-Net achieves the best performance in terms of both PSNR and SSIM, attaining an average PSNR of 48.92 dB, which substantially outperforms all competing methods. 

As shown in Table 3, CNN-based, Transformer-based, and frequency-domain methods all achieve reasonable results. However, the proposed PNG-Net, through frequency-domain prior guidance, substantially enhances the NU noise correction performance, demonstrating the effectiveness of the proposed physical guidance strategy.

To verify the generalization ability of PNG-Net, Table 4 tests the nonuniformity correction results of different methods under different noise conditions. $\sigma_{g}$, $\sigma_{s}$, $\sigma_{o}$ and $\sigma_{1/f}$ in Table 4 represent the gray-level standard deviations of Gaussian noise, stripe noise, FPS noise and 1/f noise, respectively.

To further compare the complexity of the algorithms, Table 5 shows the Params, FLOPs and inference time on the same test dataset on our NVIDIA RTX 3090 GPU. Among them, the inference time is the average inference time on the entire test dataset, the batch size during testing is 8, and the number of repeated measures on the entire test dataset is 1. All competing methods were implemented under same settings. According to the table results, the single frame inference time of PNG-Net is about 16.96 ms, indicating good real-time performance.

In order to visually demonstrate the PSD-like noise estimation results of the PSD-like noise estimator, Figure 3 shows the PSD results of the test image example and the PSD-like results output by the PSD-like noise estimator. In Figure 3, (a) to (f) respectively represent the spectrum clean image, the spectrum of noise image, the spectrum of pure noise, the PSD calculated from real noise, the noise PSD prior map learned by the PSD-like noise estimator of PNG-Net, the radial average curve of the ground true noise PSD and the estimated noise PSD. The ground true PSD is calculated from the residual between the noisy image and clean image in the synthesized test dataset.

Based on the radial average curve of the ground true noise PSD and the estimated PSD, the correlation coefficient corr between the ground true noise PSD and the estimated noise PSD was calculated to measure the similarity of the frequency-domain distribution between the two PSD graphs. The closer the corr is to 1, the more consistent the distribution of the ground true PSD and the estimated PSD. The calculated average corr of the test dataset is 0.729, indicating that the estimated PSD is close to the ground true PSD distribution, proving the effectiveness of the PSD-like noise estimator.

### 4.3. Qualitative Analysis

Figure 4 compares corrected motor infrared images. In the examples shown, several baselines leave residual patterns or smooth fine detail, whereas PNG-Net retains clearer boundaries while reducing visible noise. Figure 5 shows the corresponding restoration-error maps, computed as each corrected image minus its clean reference.

Figure 6 compares the methods on directional stripe noise. In the examples shown, PNG-Net reduces column-oriented artifacts while retaining visible scene structure. Figure 7 presents the corresponding restoration-error maps, computed as corrected images minus clean references.

### 4.4. Ablation Study

To verify the effectiveness of each individual component, a thorough ablation study was conducted. Table 6 reports the ablation results on the synthetic dataset.

From the ablation results, the following observations can be made: (1) Removing the frequency decomposition and dynamic band selector leads to a PSNR decrease of 2.65 dB, confirming the effectiveness of frequency decomposition in disentangling noise from the signal. (2) Excluding the PSD-like noise estimation and anisotropic mask causes a substantial PSNR drop of 10.15 dB, highlighting the critical importance of noise estimation and directional masking for handling directional NU noise. (3) Removing the skip-connection fusion module results in a 2.2 dB decline, indicating that the fusion module contributes positively to the denoising capability. (4) Replacing the noise-guided residual block with a plain residual block reduces PSNR by 1.49 dB, demonstrating that the noise-guided constraint effectively enhances denoising quality.

### 4.5. Limitations and Future Work

The current experiments are based on paired synthetic infrared nonuniformity data generated from a public motor thermal image dataset. Although this protocol provides clean references for PSNR, SSIM, residual, and stripe-index evaluation, it cannot fully represent all detector drift, temporal instability, lens effects, and cross-sensor variations encountered in real IRFPA systems. Future work will evaluate PNG-Net on real multi-sensor infrared sequences and include temperature-error, hot-spot localization, and downstream fault-diagnosis metrics, to achieve industrial deployment. Meanwhile, in the future, the proposed infrared image restoration framework can be combined with intelligent fault diagnosis [33,34,35]. By incorporating interpretable learning mechanisms, domain knowledge guidance, and multimodal information fusion strategies, the interpretability, robustness, and practical application value of image restoration and intelligent fault detection models can be further enhanced.

## 5. Conclusions

We proposed PNG-Net, a physics-guided network for infrared image nonuniformity correction. The method combines frequency-band decomposition, a learned PSD-like spectral noise prior, anisotropic masking, and residual restoration to address structured synthetic noise. The dynamic frequency-band selector combines features from low-, mid-, and high-frequency components. The PSD-like noise estimator and anisotropic mask provide a spectral conditioning signal for the noise-guided residual stack. Skip-connection fusion combines multi-scale features, and global residual learning produces the corrected image. PNG-Net achieved a PSNR of 48.92 dB and an SSIM of 0.990 on the synthetic test set. These results support spectral conditioning and directional feature modulation for the evaluated corruptions. Validation on real detector data remains necessary before drawing conclusions about practical IRFPA correction or industrial deployment.

## Figures and Tables

**Figure 1:**
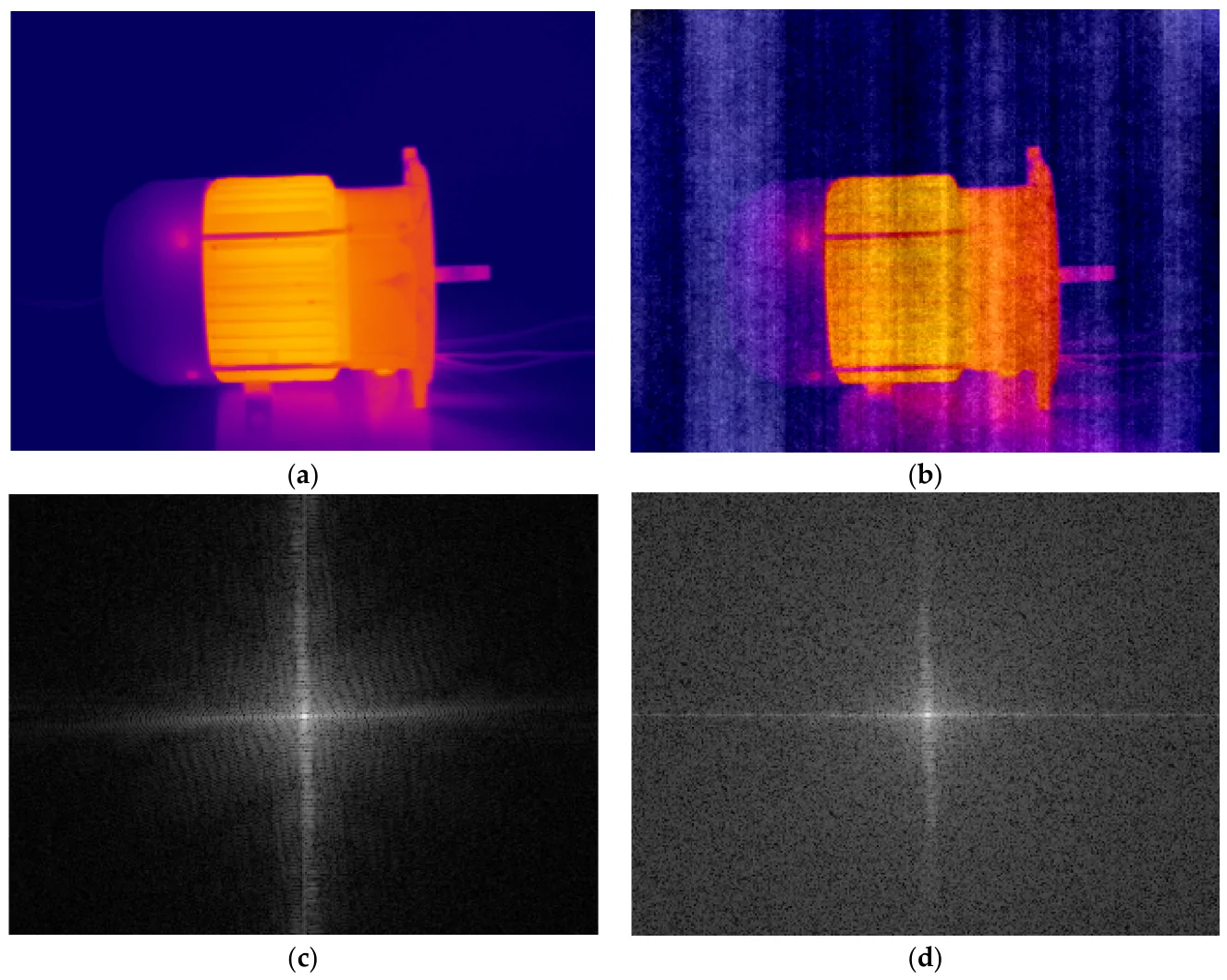
Comparison of clean and noisy images along with their corresponding spectra. Clean and noisy images are displayed in pseudo color. (**a**) Clean image; (**b**) noisy image; (**c**) spectrum of the clean image; (**d**) spectrum of the noisy image.

**Figure 2:**
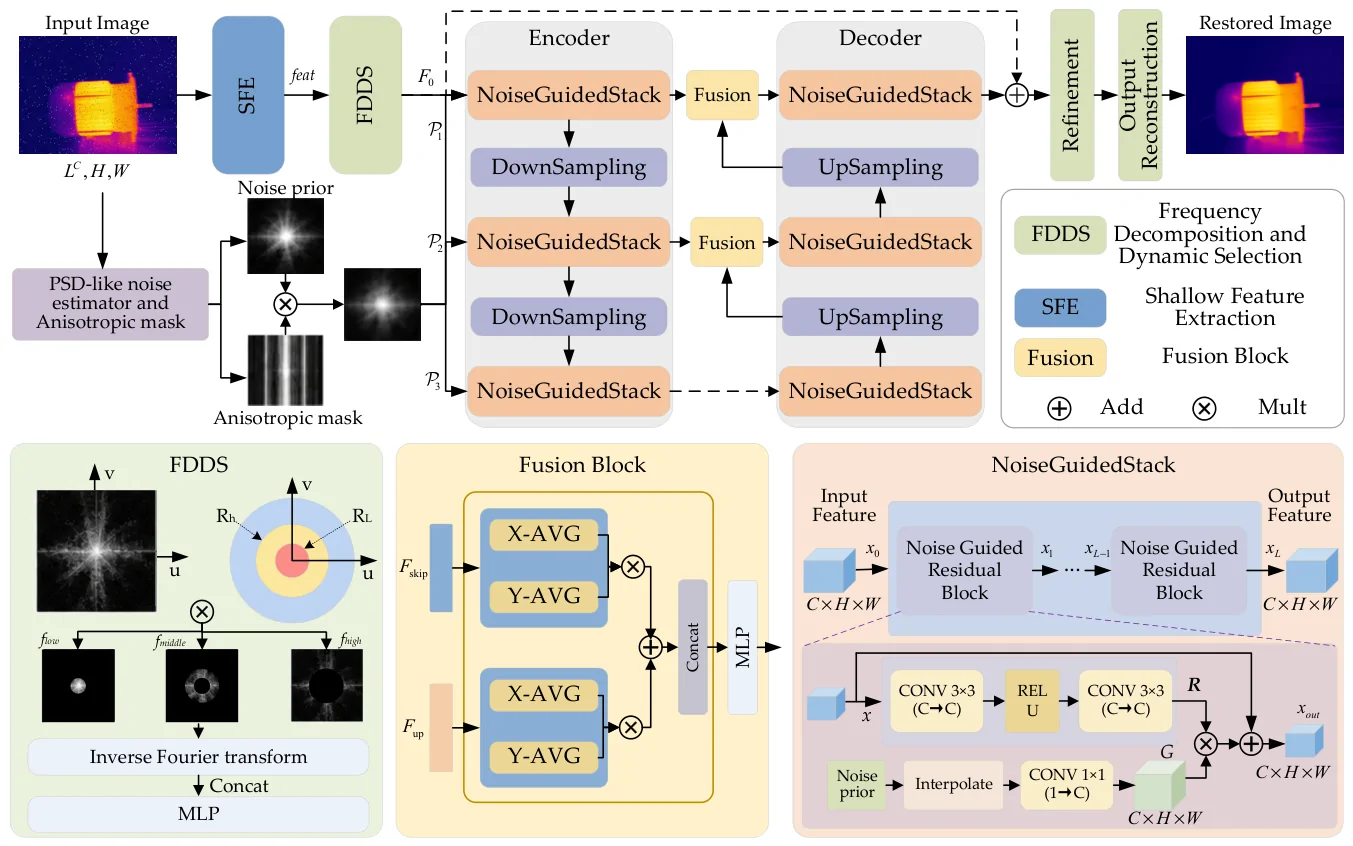
Overall architecture of the proposed PNG-Net. “...” indicates that multiple Noise Guided Residual Blocks are concatenated together.

**Figure 3:**
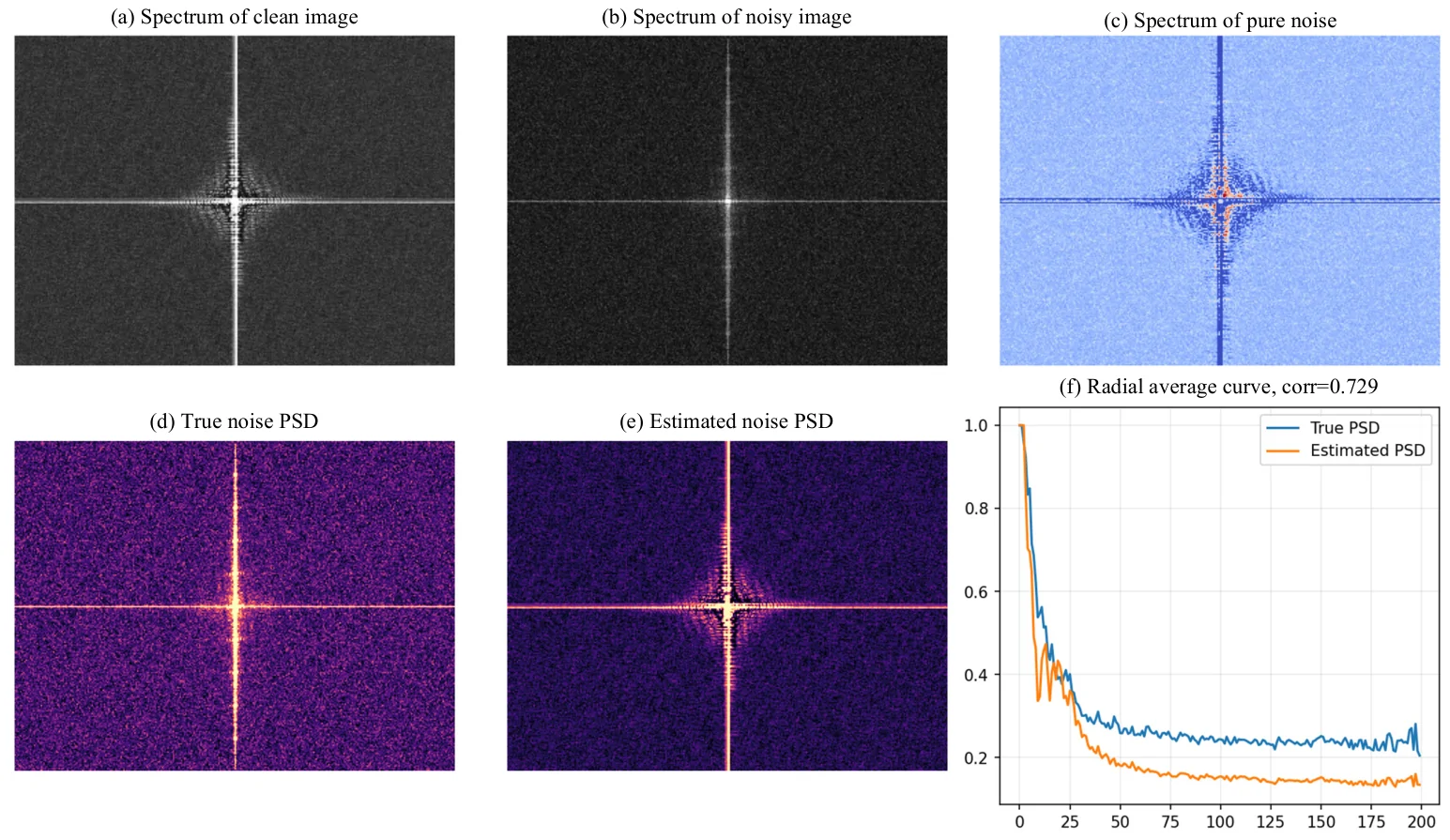
The PSD results of the test image example and the PSD-like results output by the PSD-like noise estimator. Images (**c**–**e**) are all displayed in pseudo color.

**Figure 4:**
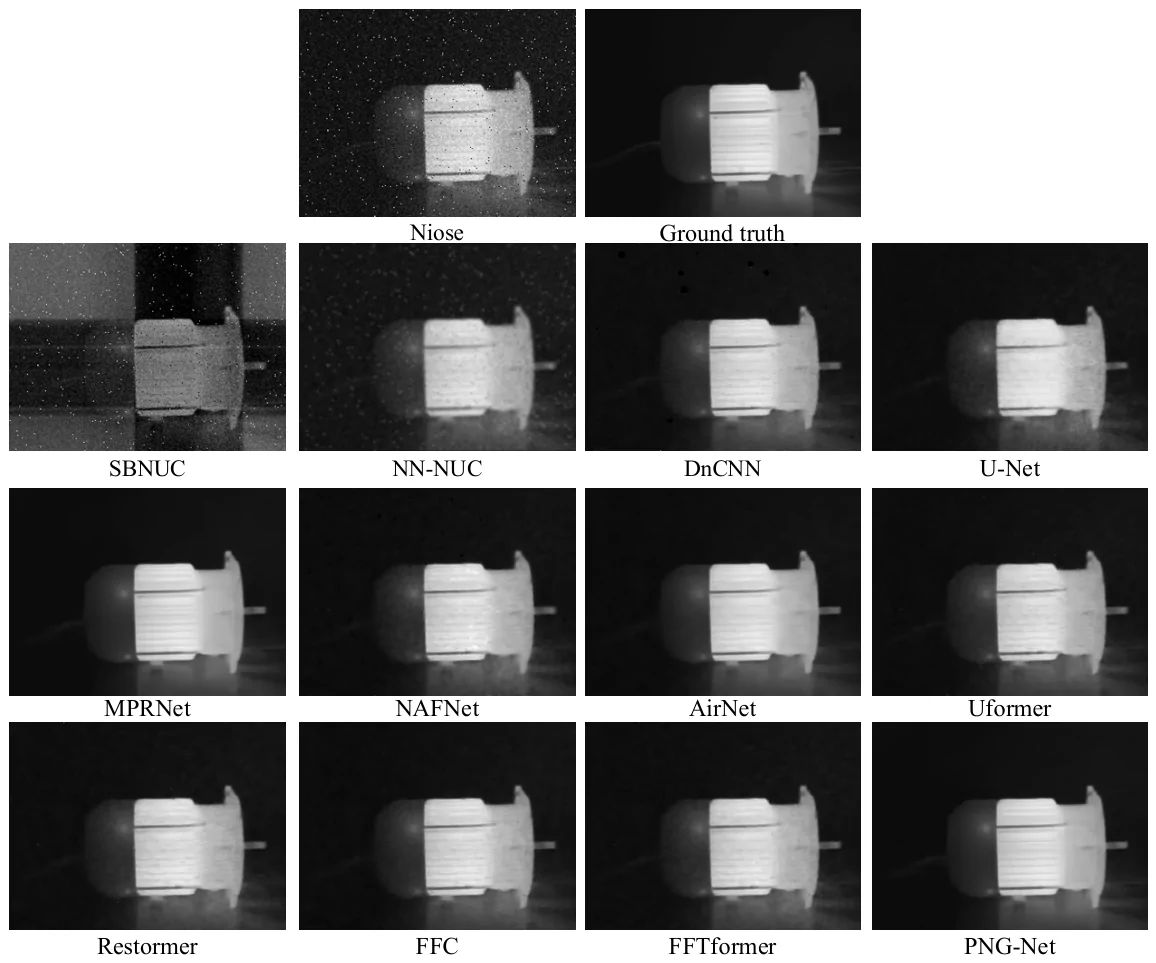
Visual comparison of denoising results from different methods.

**Figure 5:**
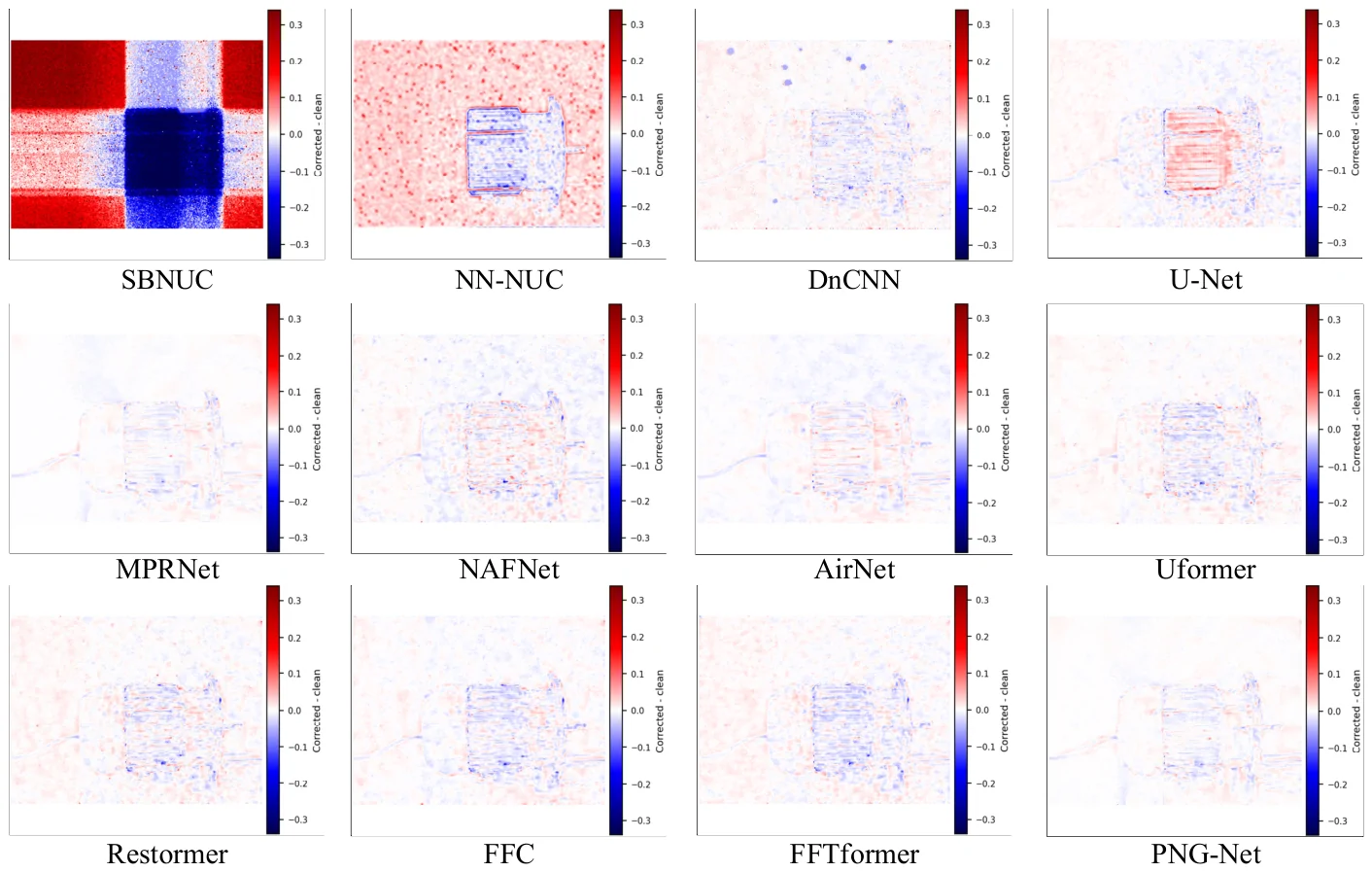
Restoration-error maps for Figure 4, computed as corrected images minus clean references.

**Figure 6:**
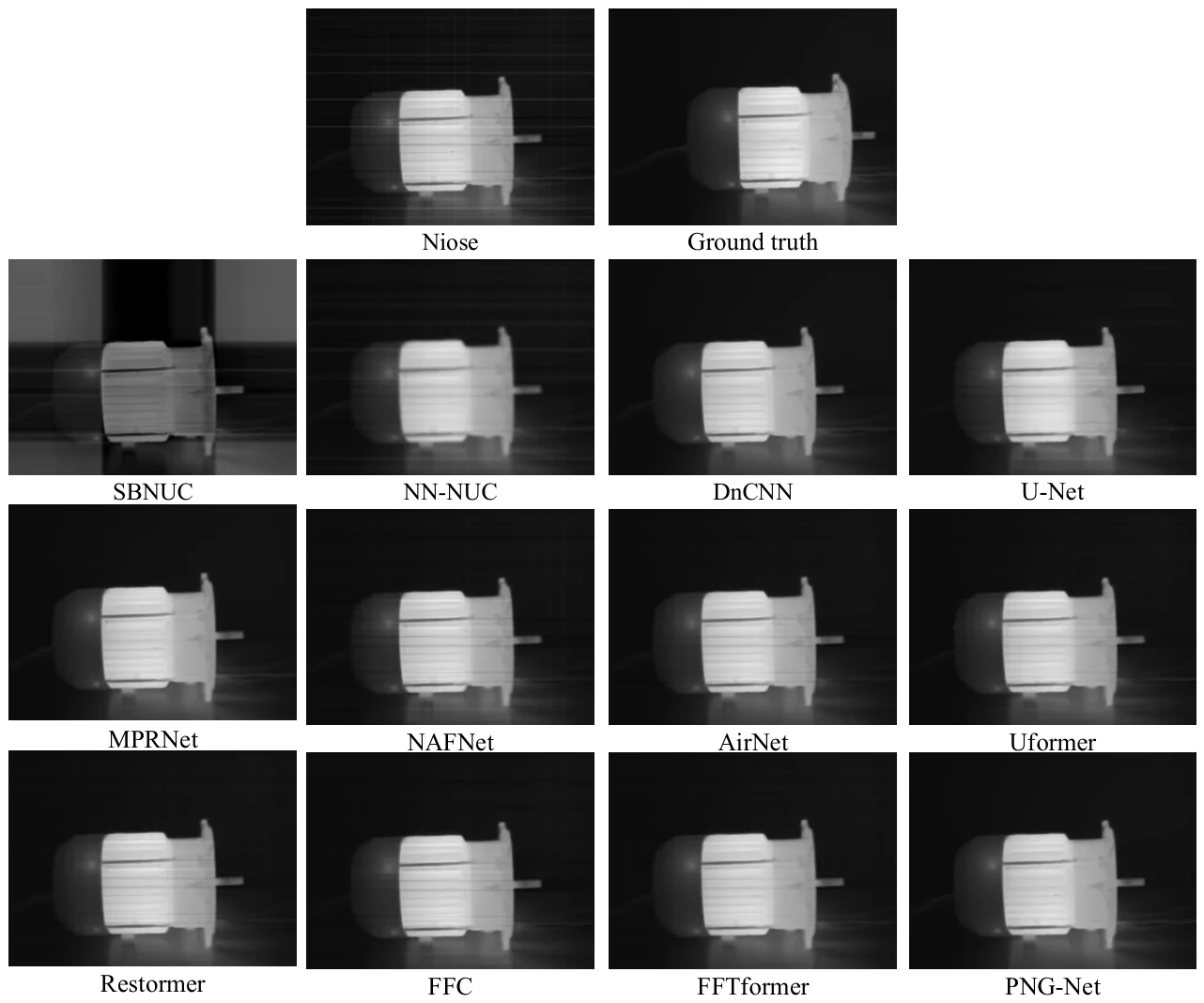
Comparison of directional noise suppression results.

**Figure 7:**
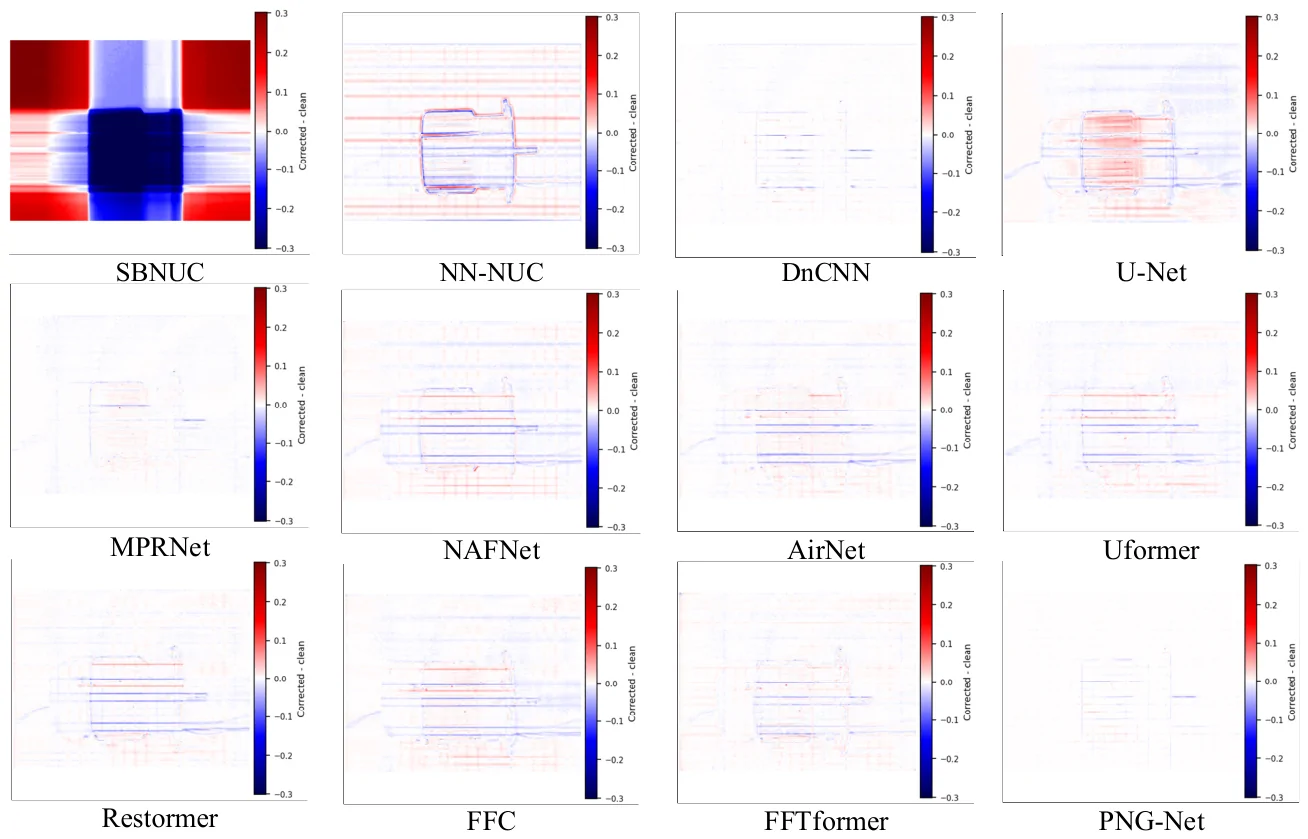
Restoration-error maps for Figure 6, computed as corrected images minus clean references.

**Table 1:** Dataset introduction. The labels “A”, “B” and “C” represent the three phases of the induction motor, while SCT refers to the number of Short-Circuited Turns within the motor. The value of SCT is expressed as a percentage in this representation.

Category of Fault	Training Set	Testing Set	Remarks
A&B50	31	7	Fault in phase A and B with 50% SCTs
A&B&C10	25	6	Fault in phase A, B and C with 10% SCTs
A&B&C30	34	8	Fault in phase A, B and C with 30% SCTs
A&C10	25	6	Fault in phase A and C with 10% SCTs
A&C30	31	7	Fault in phase A and C with 30% SCTs
A10	28	6	Fault in phase A with 10% Short-Circuited Turns (SCTs)
A30	30	7	Fault in phase A with 30% SCTs
A50	28	7	Fault in phase A with 50% SCTs
Fan	23	5	Fault in motor’s fan
Noload	20	5	Motor without load
Rotor-0	24	6	Fault in rotor
total	299	70	

**Table 2:** 8-bit gray-level ranges of standard deviation settings for different noise in the dataset.

	FPN Offset	Stripe Noise	1/f Noise	Gaussian Noise
Range	0–10	0–10	0–10	0–20

**Table 3:** Quantitative comparison table of various methods on synthetic datasets.

Method	PSNR (dB)	SSIM	SNI	RMNU	CMNU	EPI
SBNUC	22.98 ± 8.94	0.621 ± 0.262	22.80 ± 19.02	14.62 ± 12.15	17.48 ± 14.65	0.64 ± 0.29
NN-NUC	34.99 ± 6.94	0.872 ± 0.118	3.23 ± 4.41	2.48 ± 2.77	1.86 ± 3.55	0.66 ± 0.24
DnCNN	44.16 ± 9.68	0.967 ± 0.043	2.29 ± 4.88	1.62 ± 3.14	1.57 ± 3.76	0.75 ± 0.26
U-Net	40.79 ± 7.27	0.967 ± 0.035	3.11 ± 4.50	2.05 ± 2.87	2.31 ± 3.49	0.74 ± 0.25
MPRNet	48.00 ± 9.46	0.984 ± 0.036	2.19 ± 4.79	1.51 ± 3.07	1.57 ± 3.68	0.86 ± 0.20
NAFNet	42.45 ± 6.59	0.973 ± 0.027	2.71 ± 4.57	2.07 ± 2.91	1.56 ± 3.62	0.70 ± 0.28
AirNet	44.29 ± 7.69	0.979 ± 0.031	2.53 ± 4.71	1.93 ± 3.01	1.44 ± 3.69	0.78 ± 0.26
Uformer	43.50 ± 7.98	0.974 ± 0.038	2.47 ± 4.70	1.87 ± 2.99	1.49 ± 3.68	0.75 ± 0.27
Restormer	43.71 ± 7.03	0.977 ± 0.027	2.47 ± 4.55	1.87 ± 2.88	1.47 ± 3.58	0.74 ± 0.27
FFC	43.28 ± 6.98	0.977 ± 0.029	2.57 ± 4.36	1.93 ± 2.78	1.57 ± 3.43	0.73 ± 0.26
FFTformer	44.51 ± 7.28	0.979 ± 0.029	2.27 ± 4.57	1.65 ± 2.88	1.48 ± 3.57	0.75 ± 0.26
**PNG-Net**	**48.92 ± 8.88**	**0.990 ± 0.016**	**1.12 ± 3.07**	**0.72 ± 1.88**	**0.86 ± 2.43**	**0.93 ± 0.12**

**Table 4:** Test results of nonuniformity correction under different noise conditions.

Method	$\sigma_{g}$ **= 10** $\sigma_{s}$ **= 5** $\sigma_{o}$ **= 0~10** $\sigma_{1/f}$ **= 0~10**	$\sigma_{g}$ **= 20** $\sigma_{s}$ **= 10** $\sigma_{o}$ **= 0~10** $\sigma_{1/f}$ **= 0~10**	$\sigma_{g}$ **= 30** $\sigma_{s}$ **= 15** $\sigma_{o}$ **= 0~10** $\sigma_{1/f}$ **= 0~10**	$\sigma_{g}$ **= 40** $\sigma_{s}$ **= 20** $\sigma_{o}$ **= 0~10** $\sigma_{1/f}$ **= 0~10**	$\sigma_{g}$ **= 0~20** $\sigma_{s}$ **= 0~10** $\sigma_{o}$ **= 20** $\sigma_{1/f}$ **= 0~10**	$\sigma_{g}$ **= 0~20** $\sigma_{s}$ **= 0~10** $\sigma_{o}$ **= 0~10** $\sigma_{1/f}$ **= 20**
	PSNR(dB) /SSIM	PSNR(dB) /SSIM	PSNR(dB) /SSIM	PSNR(dB) /SSIM	PSNR(dB) /SSIM	PSNR(dB) /SSIM
SBNUC	21.83/0.39	19.51/0.22	17.69/0.13	16.33/0.09	21.80/0.40	19.52/0.22
NN-NUC	33.59/0.84	28.67/0.66	25.28/0.51	23.02/0.42	34.64/0.84	29.84/0.65
DnCNN	33.51/0.86	29.68/0.72	27.15/0.59	25.15/0.48	34.60/0.87	30.51/0.73
U-Net	34.65/0.93	31.58/0.86	28.99/0.81	26.85/0.72	35.66/0.93	32.57/0.87
MPRNet	30.31/0.81	27.99/0.72	25.64/0.62	23.73/0.55	28.05/0.73	26.44/0.62
NAFNet	31.96/0.83	27.25/0.61	25.88/0.52	25.04/0.48	34.66/0.88	30.01/0.71
AirNet	34.05/0.87	28.92/0.67	26.17/0.50	24.45/0.37	35.87/0.89	30.48/0.70
Uformer	32.37/0.85	28.16/0.64	26.19/0.52	24.61/0.43	33.97/0.87	29.11/0.69
Restormer	32.22/0.86	28.23/0.69	25.94/0.55	24.05/0.45	34.55/0.88	29.73/0.70
FFC	33.65/0.88	28.98/0.70	26.94/0.59	25.36/0.51	34.98/0.90	29.88/0.72
FFTformer	30.31/0.78	24.92/0.55	22.97/0.45	21.34/0.37	33.86/0.84	26.93/0.58
**PNG-Net**	**41.45/0.97**	**38.36/0.96**	**36.16/0.94**	**34.22/0.91**	**38.65/0.95**	**32.92/0.88**

**Table 5:** Model complexity and single-image inference time on 240 × 320 infrared images.

Method	Params (M)	FLOPs (G)	Time (ms/Image)
DnCNN	0.313	47.91	10.37
U-Net	1.050	31.92	5.18
MPRNet	2.314	149.17	25.69
NAFNet	0.097	12.22	9.94
AirNet	0.403	51.72	19.14
Uformer	0.300	10.04	22.55
Restormer	0.086	12.78	44.94
FFC	0.260	34.14	10.71
FFTformer	0.221	33.17	45.26
PNG-Net	2.405	62.04	16.96

**Table 6:** Results of ablation experiment.

Configuration	PSNR (dB)	SSIM
Full model	**48.92**	**0.990**
Without frequency decomposition and dynamic selection	46.27	0.984
Without frequency decomposition	48.19	0.989
Without dynamic selection	48.78	0.989
Without PSD-like noise estimator and anisotropic mask	38.77	0.909
Without PSD-like noise estimator	46.69	0.985
Without anisotropic mask	48.63	0.989
Without skip-connection fusion module	46.72	0.986
Remove noise-guided residual block (replaced with plain residual block)	47.43	0.982

## Data Availability

The clean infrared motor images are derived from the publicly available motor thermal fault dataset reported in Ref. [29]. To ensure reproducibility, the author can provide the synthetic corruption protocol, train/test split, configuration files, and generated correction results upon request.
